# The First Mitogenomes of the Subfamily Odontiinae (Lepidoptera, Crambidae) and Phylogenetic Analysis of Pyraloidea

**DOI:** 10.3390/insects12060486

**Published:** 2021-05-24

**Authors:** Mujie Qi, Huifeng Zhao, Fang Yu, Aibing Zhang, Houhun Li

**Affiliations:** 1 College of Life Sciences, Nankai University, Tianjin 300071, China; qimujie@nankai.edu.cn; 2 Hebei Key Laboratory of Animal Diversity, College of Life Science, Langfang Normal University, Langfang 065000, China; zhaohf@lfnu.edu.cn; 3 College of Life Sciences, Capital Normal University, Beijing 100048, China; zhangab2008@mail.cnu.edu.cn; 4 Jiangsu Key Laboratory of Biofunctional Molecule, School of Life Sciences, Chemistry & Chemical Engineering, Jiangsu Second Normal University, Nanjing 211200, China; yufang@jssnu.edu.cn

**Keywords:** mitogenome, Pyraloidea, Crambidae, Odontiinae, phylogeny

## Abstract

**Simple Summary:**

The Odontiinae is a small group in the Pyraloidea comprised of 388 species in 88 genera, but externally, these moths are diverse, including heterogeneous maculation and a size range from 9 to 50 mm in total wingspan. The monophyly of Pyraloidea and the two families (Pyralidae and Crambidae) is well supported by phylogenetic analyses based on morphology and molecular data of multiple nuclear genes. However, only a few mito-phylogenetic analyses have been conducted and no mitogenome of Odontiinae species has been reported. Three complete mitogenomes of odontiine species were sequenced and analyzed for the first time herein. The results showed that Odontiinae mitogenomes shared similar genomic characters with other Pyraloidea. The phylogenetic analyses based on 13 PCGs of mitogenomes confirmed the monophyly of Odontiinae and its position within Crambidae.

**Abstract:**

The complete mitochondrial genomes of three species of Odontiinae were newly sequenced: *Dausara latiterminalis* Yoshiyasu, *Heortia vitessoides* (Moore), and *Pseudonoorda nigropunctalis* (Hampson). These circular and double-stranded mitogenomes vary from 15,084 bp to 15,237 bp in size, including 13 protein-coding genes (PCGs), two ribosomal RNA genes (rRNAs), and 22 transfer RNA genes (tRNAs) and an A + T-rich region. The nucleotide composition indicated a strong A/T bias. Most PCGs are initiated with an ATN codon and terminated by a codon of TAR. All tRNAs could be folded into the clover-leaf structure with the exception of trnS1 (AGN), in which the dihydrouridine (DHU) arm formed a simple loop, and the motif ‘ATAG’ and ‘ATTTA’ in the A + T-rich region was also founded. The phylogenomic analyses covering Odontiinae + 11 subfamilies of Pyraloidea were conducted. Similar topologies were generated from both Bayesian inference (BI) and maximum likelihood (ML) analyses based on the nucleotide and amino acid sequence data. There was some discrepancy in the sister-group relationship of Odontiinae and Glaphyriinae, and the relationships among the subfamilies in the ‘CAMMSS clade’ of the Crambidae. The results of this study suggest that mitogenomic data are useful for resolving the deep-level relationships of Pyraloidea and the topologies generated from amino acid data might be more realistic and reliable. Moreover, more mitogenomic taxon sampling and larger scale analyses with more genes or a combination of mitogenomic and nuclear genes are needed to reconstruct a comprehensive framework of the pyraloid phylogeny.

## 1. Introduction

Within Lepidoptera, the Pyraloidea is one of the largest superfamilies that includes two families—Pyralidae and Crambidae. This superfamily, found on all continents except Antarctica, comprises about 16,000 named species worldwide [1,2]. As one of the small subfamilies of Pyraloidea, Odontiinae is comprised of 388 species in 88 genera, and it is numerous in eremic habitats of all major biogeographic regions except New Zealand. Many odontiine species are important pests, and their larvae are leaf miners and folders, flower and bud feeders, and fruit borers [3,4,5]. They usually cause serious damage to medicinal plants and fruit trees such as the incense trees (*Aquilaria sinensis*) and olive trees (*Canarium album*) in tropical and subtropical regions [6,7]. Historically, the relationships among Odontiinae, Glaphyriinae, Noordinae, Evergestinae and Cathariinae have been controversial for a long time until the landmark studies conducted by Regier et al. [8] and Léger et al. [4] which discussed the phylogeny of Pyraloidea including Odontiinae based on several nuclear genes combined with a single mitochondrial gene COI. However, a mitogenome-based investigation about the relationships among Odontiinae and the other subfamilies of Pyraloidea has never been discussed.

The typical arthropod mitochondrial genome (mitogenome) is a circular, double-stranded molecule which encodes 37 genes, including 13 protein-coding genes (PCGs), two ribosomal RNA genes (rRNAs), 22 transfer RNA genes (tRNAs), and an A + T-rich region [9,10]. Due to cellular abundance, an absence of introns, rapid evolutionary rate, and a lack of extensive recombination, mitogenome sequences can be easily amplified and have been proven to be a useful source that has been extensively employed in systematics, population genetics and evolutionary biology in the past decade [11,12,13,14,15]. In recent years, mitochondrial genomes from different subfamilies of Pyraloidea have been obtained, and several mitogenome-based investigations with respect to the phylogeny of this superfamily have been performed [16,17,18,19,20,21]. Unfortunately, a mitochondrial genome of the subfamily Odontiinae has remained unknown. Moreover, 50+ mitogenomes of Pyraloidea are a tiny part comparing with 16,000 named species of the superfamily. Therefore, more mitogenomes of pyraloids are needed to be sequenced and included in phylogenetic analyses.

To begin to rectify some of the above issues, three complete mitogenomes of odontiine species, including *Dausara latiterminalis* Yoshiyasu, *Heortia vitessoides* (Moore), and *Pseudonoorda nigropunctalis* (Hampson) were sequenced and annotated for the first time. In addition, a comparative analysis was conducted to reveal the genomic organization, nucleotide composition, codon usage, and tRNA secondary structure. Moreover, we used the newly sequenced mitochondrial genomes of this study and mitogenomes available online to reconstruct a phylogenetic tree of Pyraloidea to better understand their evolutionary history and relationships within Pyraloidea.

## 2. Materials and Methods

### 2.1. Specimen Collection and Genomic DNA Extraction

Three odontiine species were collected by light traps in China, Malaysia and Laos (Supplementary Table S1), respectively, and were preserved in 99.5% ethanol during fieldwork. They were then stored at −50 centigrade degree environment in the Insect Collection of Nankai University (NKU), Tianjin, China.

All the above specimens were identified based on the morphological characters. Thorax and legs were used to extract genomic DNA with the DNeasy tissue kit (Qiagen, Hilden, Germany) according to the manufacturer’s protocol. DNA concentrations were measured with a DeNovix DS-11 Spectrophotometer, DNA integrity was examined with agarose gel electrophoresis by 0.5 × TBE (Tris base, Boric acid and EDTA) buffer with 3 Volt/centromere for 45 min.

### 2.2. High throughout Sequencing

All three odontiine genomic DNA were qualified for high throughput sequencing (HTS). The genomic DNA was fragmented to 350–500 bp by Covaris S220 Focused Ultrasonicator (Covaris, MA, USA). The sequence libraries were constructed using TruSeq DNA LT Sample Preparation Kit (Illumina, Inc., San Diego, CA, USA). After repairing the blunt ends, adenylating 3′ ends and ligating adapters, the fragmented DNA were enriched. Those libraries were pooled and sequenced by an Illumina Hiseq X10 platform. The raw data were filtered as follows: (1) the adapters were removed; (2) the reads that contained more than five Ns were removed; (3) 4 base slide windows were performed, and the reads or the averaged Phred value of less than Q20 were removed; (4) after the above steps, the reads shorter than 75 bp or the Phred values less than Q15 were removed. Finally, approximately 40 Gb clean data of paired-end reads of 150 bp length were generated.

### 2.3. Data Assemble and Annotation

The raw data were assembled by MitoZ v2.4 [22] with the ‘all’ option, which finalized data assembly, de novo assembly, genome annotation, and visualization only within one step. The assembled circular mitogenomes were reordered trnM as a start gene with the script ‘Mitogenome_reorder.py’ in MitoZ. All calculation was performed in the high-performance computing platform in Capital Normal University.

As for the sequence length of the control region, less than 600 bp is not annotated by mitoZ, and the known length of the A + T-rich region in most lepidopteran species ranges from 280 to 500 bp [20,21,23,24,25]. The annotation of the three mitochondrial genomes was also performed by the MITOS2 online server with default parameters (http://mitos2.bioinf.uni-leipzig.de/index.py, accessed on 30 March 2021). The A + T-rich region was ensured according to the results of MITOS2 and the other available mitochondrial genomes of Crambidae [20].

### 2.4. Statistics of the Odontiine Mitochondrial Genomes

Nucleotide composition and relative synonymous codon usage (RSCU) of the odontiine mitogenomes were calculated in MEGA 5 [26]. Nucleotide compositional skew was calculated according to the formulas: AT skew = [A − T]/[A + T], GC skew = [G − C]/[G + C]) [27].

### 2.5. Phylogenetic Analyses

To investigate the phylogenetic implications of the mitogenomes of the three species in Pyraloidea, we reconstructed the subfamily-level relationships within Pyraloidea using three different datasets of the 13 protein coding genes (PCG) and two inference methods.

The mitogenomic phylogeny of Pyraloidea was reconstructed with 40 ingroups (37 online data and 3 newly produced data in this study) and 5 outgroups (Table 1). The three datasets are PCG123 (13 PCGs including all codon positions), PCG12 (13 PCGs without third codon positions) and AA (amino acid of 13 PCGs). Bayesian inference (BI) and maximum likelihood (ML) methods were used to reconstruct phylogenetic trees.

For PCG123 and PCG12 datasets, the best DNA model based on Akaike information criterion (AIC) was performed using jModeltest 2.1.7 [52] (Table S2), and those selected models were used by BI with software MrBayes 3.2.6 [53]. To ensure that the average standard deviation of split frequencies was less than 0.01, eight million generations were run with sampling every 1000 generations. Node support was assessed by posterior probabilities (PPs). The ML analyses were performed using IQ-TREE v2.1.2 [54], selecting the best model and constructing phylogenetic trees automatically using 1000 non-parametric bootstrap replicates (BS) and SH-aLRT test with an unpartitioned strategy (‘-m MFP -b 1000 alrt 1000′), whilst other settings were default.

To mitigate the possible effects of base-composition bias and among-site rate heterogeneity, the AA dataset was analyzed using Phylobayes v4.1c [55], and the MtZoa model, which fit the mitogenomic phylogenetic analyses in metazoan groups, was chosen to perform the BI analyses [56]. Two independent MCMC chains in Phylobayes were run until convergence (maxdiff < 0.1 and minimum effective size > 50). As for the ML analysis, the dataset AA was performed by IQ-TREE v2.1.2 with the parameters ‘-m MtZoa + F + I + G4 -b 1000 -alrt 1000′. Tracer v1.6 [57] was used to check the likelihoods of all parameters of BI analyses of the three datasets to ensure the effective sample size (ESS) values greater than 200. The consensus tree was calculated by discarding the first 25% trees. To verify the consistencies of the topologies, both BI and ML analyses were repeated three times, and the phylogenetic trees were visualized by Figtree v.1.4.3 [58].

## 3. Results and Discussion

### 3.1. Mitogenome Organization and Nucleotide Composition of Odontiinae

**Figure 1:**
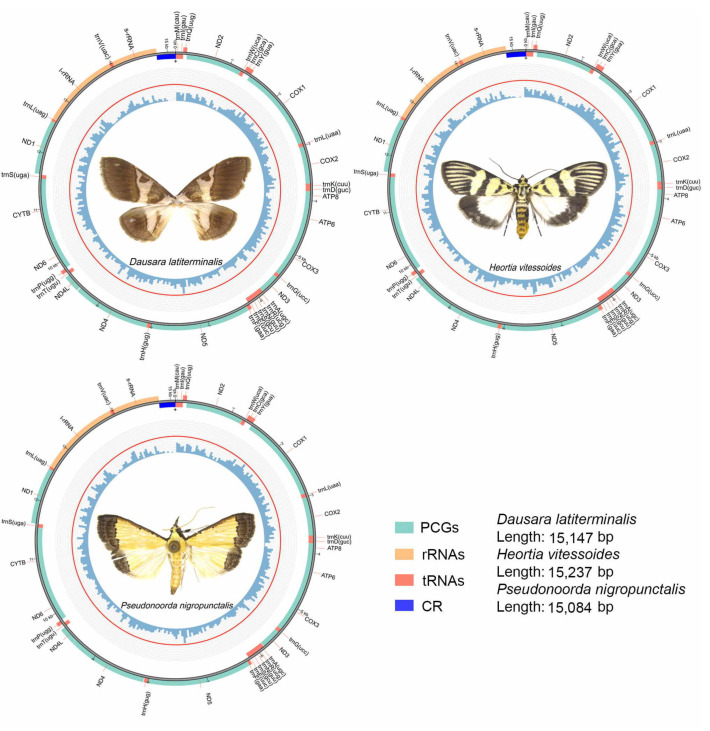
Complete mitochondrial genomes of three species for Odontiinae. The inner circle indicates the GC content, the outer circle shows the arrangement of the genes: green for the CDS, red for tRNAs, orange for rRNAs, and blue for control region.

The annotations for the mitogenomes of the three odontiine species are shown in Table 2, and the circular maps of the mitogenomes of the three species are shown in Figure 1. The complete mitogenomes of the three species were investigated here, and all were found to be composed of circular double stranded molecules with mildly varying sizes. Each mitogenome contains the typical set of 37 genes, including 13 typical protein-coding genes (PCGs), 22 transfer RNA genes (tRNAs), two ribosomal RNA genes (rRNAs) and an A + T rich area. The majority strand (J-strand) encoded 23 genes (9 PCGs, 14 tRNAs), while the remaining genes were located on the minority strand (N-strand) (four PCGs, eight tRNAs and two rRNAs) (Figure 1, Table 2). The sizes were as follows: *Dausara latiterminalis* 15,147 bp, *Heortia vitessoides* 15,237 bp, and *Pseudonoorda nigropunctalis* 15,084 bp. Gene orders of these three Odontiinae species were identical to those of other species reported in the Crambidae [35,37], specifically in reference to the tRNA gene cluster trnI–trnQ–trnM that was rearranged to trnM–trnI–trnQ.

In the whole mitogenomes of the three Odontiinae species, the nucleotide composition is as shown in Table S3. It indicated a strong A and T bias, and the A + T% content ranged from 80.6% (in *Dausara latiterminalis* and *Heortia vitessoides*) to 81.0% (in *Pseudonoorda nigropunctalis*). Comparing the AT content of the whole mitogenome, control region, PCGs, tRNAs, and rRNAs, the A + T-rich region was the highest while the PCGs was the lowest for all the three species of Odontiinae (Table S3). The AT skew of the whole mitogenome within the three odontiine species ranged from −0.012 to −0.003, and GC skew ranged from −0.201 to −0.172, which was consistent with that of several previously reported pyraloid species [35,49,59,60].

### 3.2. Protein-Coding Genes of Odontiinae

**Figure 2:**
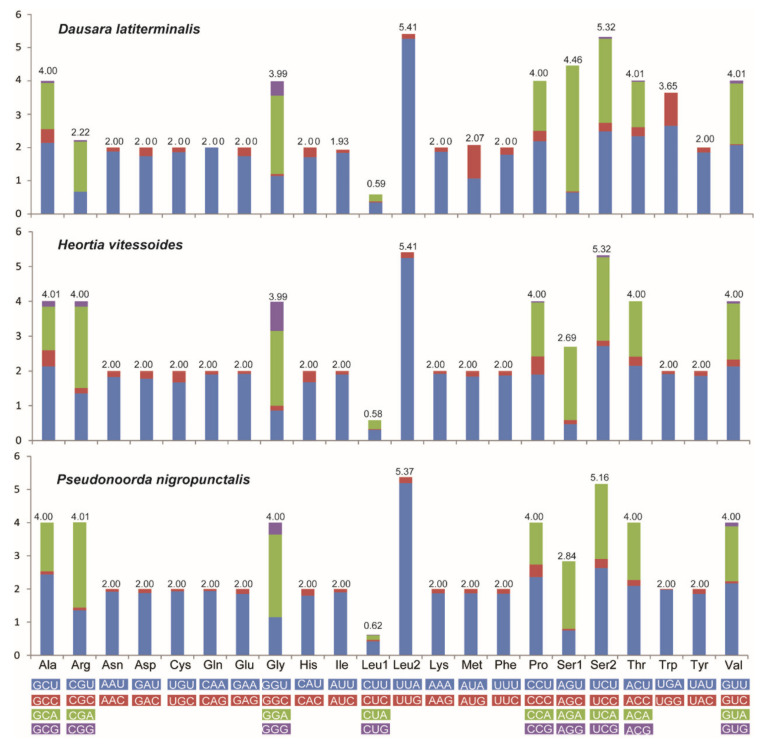
Relative synonymous codon usage (RSCU) in the PCGs of the new sequenced Odontiinae mitogenomes. Codon families are indicated below the X axis.

The PCGs within species of Odontiinae ranged from 162 bp (ATP8) to 1752 bp (ND5) in size, and the total PCGs lengths ranged from 11,268 bp to 11,313 bp. The three mitogenomes of Odontiinae exhibited similar start and stop codons as follows (Table 2): all the initiation codons of PCGs were ATN, and ATT was the most frequently used start codon for ND2, ATP8, ND3, and ND5; ATG was the most frequently used for COII, ATP6, COIII, ND4, ND4L, Cob and ND1, except for the COI which started with CGA. Except for COII which terminated with a single T residue, twelve PCGs were terminated by the standard stop codon TAR, and TAA was the most frequently used codon. Truncated termination codons are commonly used in metazoan mitogenomes and are modified by the post-transcriptional poly-adenylation to a complete TAA stop codon [61]. The RSCU values of the three odontiine species are shown in Figure 2. The codons UUA-Leu2, UCU-Ser2, GGA-Gly and UCA-Ser2 were the most commonly used in the Odontiinae mitogenome, while AGC-Ser1 was not present in these PCGs.

### 3.3. RNA Genes of Odontiinae

**Figure 3:**
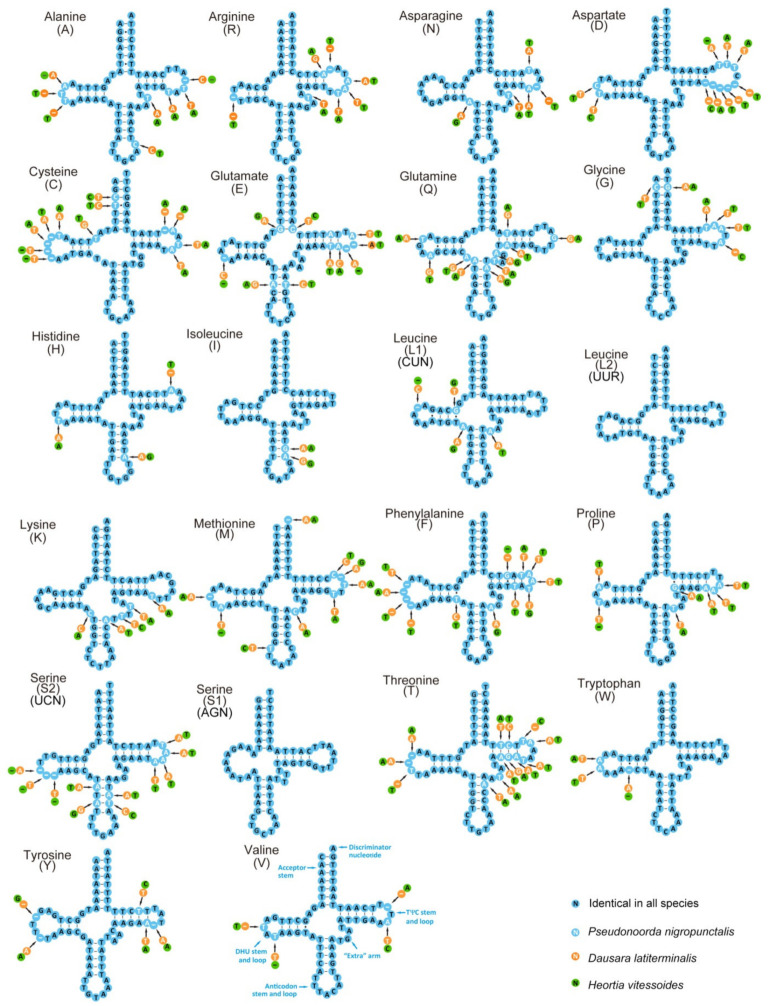
Predicted secondary structure for the tRNAs of *Dausara latiterminalis*, *Heortia vitessoides*, and *Pseudonoorda nigropunctalis*. The tRNAs are labeled with the abbreviations of their corresponding amino acids. Dashes indicate the Watson–Crick base pairs, and dots indicate the wobble GT, TT, GA pairs.

The 22 transport RNA (tRNA) genes of the three odontiine species were discovered (Figure 3), and the entire lengths of the three mitogenomes was 1452 bp in *Pseudonoorda nigropunctalis*, 1463 bp in *Dausara latiterminalis*, and 1471 bp in *Heortia vitessoides*. The size of the 22 tRNA genes ranged from 63 to 71 bp (Table 2). Among them, 21 tRNAs could be folded into the typical clover-leaf structure, except for trnS (AGN), which lost a dihydrouridine (DHU) arm (Figure 3). This phenomenon is common in Pyraloidea and in other insect mitogenomes [50,62,63,64,65]. The secondary structures comprised of the anticodon loop (7 nt), anticodon stem (5 bp), and the acceptor stem (7 bp) were conserved in length, while the length of DHU (3–4 bp) and TψC (4–5 bp) stems was variable, except for trnS1. Additionally, the identified unmatched base pairs in different stems of tRNAs are shown in Figure 3, and these mismatched nucleotides might be restored during the post-transcriptional editing processes [66].

As for the ribosomal RNA (rRNA) of the three species of Odontiinae, both of l-rRNA (rrnL) and s-rRNA (rrnS) genes were encoded on the N-strand, and the rrnL ranged from 1364 bp (in *Heortia vitessoides*) to 1369 bp (in *Dausara latiterminalis* and *Pseudonoorda nigropunctalis*) in length, while the rrnS ranged from 780 bp (in *Heortia vitessoides*) to 784 bp (in *Pseudonoorda nigropunctalis*). Both of the ribosomal RNAs showed a positive AT skew and negative GC skew, and the A + T% was about 84% in rrnL while about 86% in rrnS among the three odontiine species.

### 3.4. A + T-Rich Region of Odontiinae

**Figure 4:**
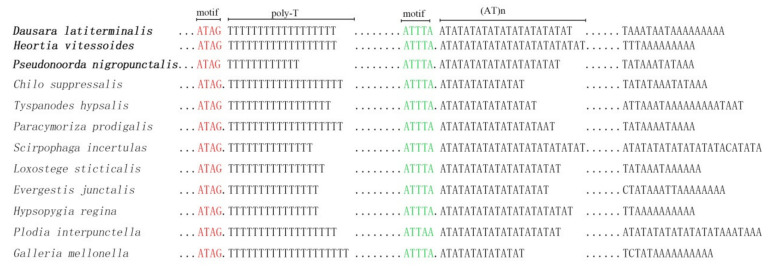
Conserved features present in the A + T-rich region of Odontiinae and other Pyraloidea. Schematic illustration of the A + T-rich region from the three newly determined mitogenomes. The conserved motifs ATAG and ATTTA are marked as red and green, respectively. Dots indicate omitted sequences, and the number of dot is not proportional to nucleotide number of the corresponding part. New sequenced species in this study were in bold.

In mitogenome, the largest non-coding region is normally the A + T-rich region (also called the control region). The A + T-rich region of odontiine mitogenomes are located between the rrnS and trnM genes, and the length was 280 bp in *Pseudonoorda nigropunctalis*, 335 bp in *Dausara latiterminalis*, and 347 bp in *Heortia vitessoides*. These lengths were similar to those in other mitogenomes of Pyraloidea (about 340 bp on average) [18]. The A + T% content was 95.2% in *Dausara latiterminalis*, 96.8% in *Heortia vitessoides*, and 96.4% in *Pseudonoorda nigropunctalis*. The comparison of the control regions of the representatives of 10 subfamilies of Pyraloidea is shown in Figure 4. Although the length of the control regions varied in different subfamilies, several conserved elements were discovered: the motif ATAG and the following large poly-T stretch, the motif ATTTA and the following microsatellite structures (AT)n, and the poly-A stretch. These conserved blocks were considered to play a key role in controlling the replication and transcription of the mitogenome [67].

### 3.5. Phylogenetic Analysis

**Figure 5:**
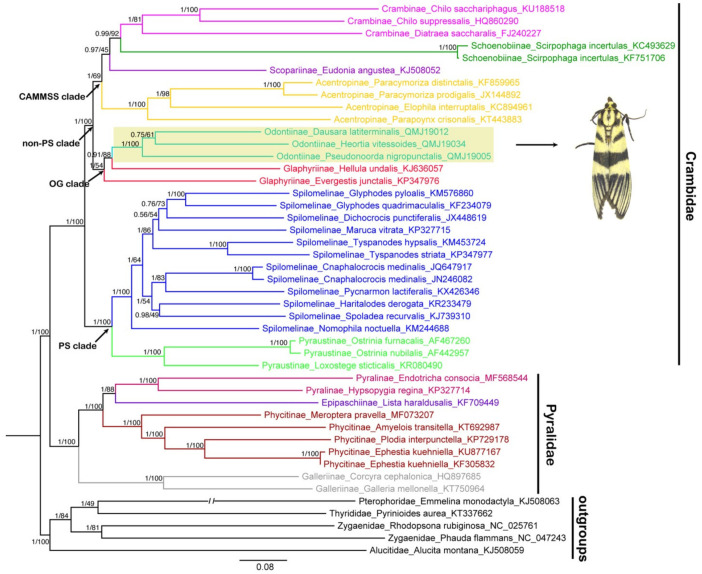
Phylogenetic trees constructed by BI/ML methods based on the dataset of PCG123, both BI and ML analyses show the same topology. The values above the branches are Bayesian posterior probabilities (PPs) and bootstrap support values (BS). Odontiinae clade is highlighted and the photo is an adult *Heortia vitessoides*. Double slash indicates a shortened clade.

**Figure 6:**
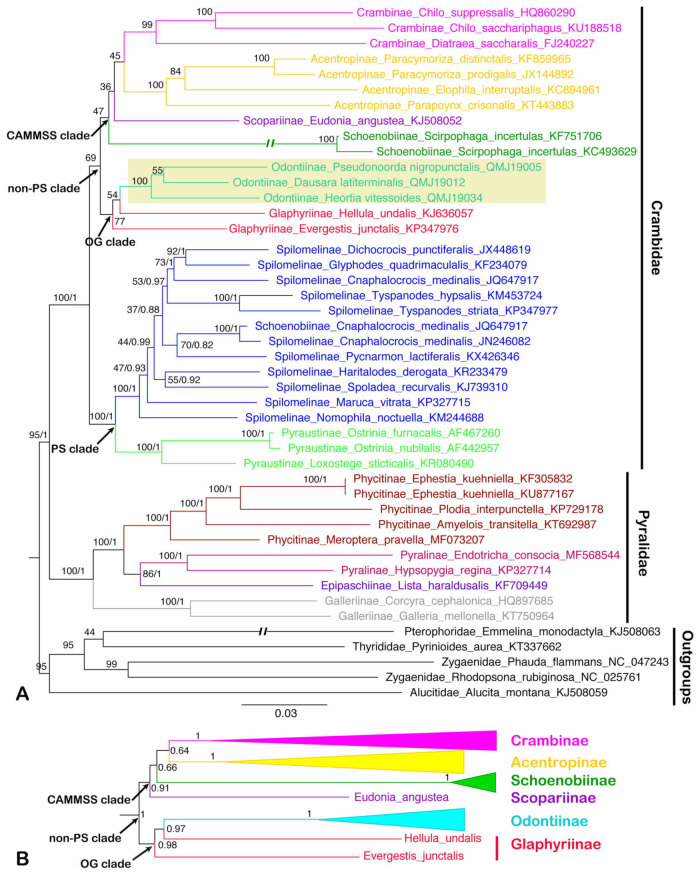
Phylogenetic trees constructed by ML/BI methods based on the dataset of PCG12, both BI and ML analyses show the similar topology except for the ‘non-PS clade’: (**A**) the ML tree of dataset PCG12; (**B**) part of the BI tree (the ‘non-PS clade’) of dataset PCG12 which is not identical to the ML tree. The values around the nodes are Bayesian bootstrap support (BS) and posterior probabilities (PPs) values. Odontiinae clade was highlighted. Double slash indicates a shortened clade.

**Figure 7:**
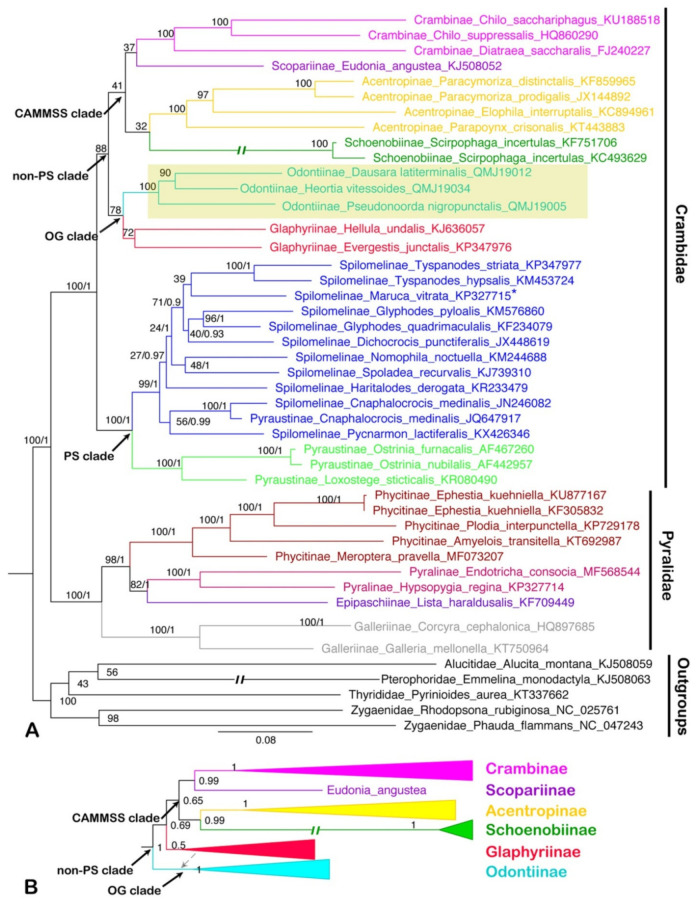
Phylogenetic trees constructed by ML/BI methods based on the dataset of AA, both BI and ML analyses show the similar topology except for the ‘non-PS clade’: (**A**) the ML tree of dataset AA; (**B**) part of the BI tree (the ‘non-PS clade’) of dataset AA which is not identical to the ML tree. The values around the nodes are Bayesian bootstrap support (BS) and posterior probabilities (PPs) values. Odontiinae clade was highlighted. Double slash indicated a shortened clade. Dotted line showed the placement of Glaphyriinae in ‘OG clade’. Asterisk showed that the position of that species was changed to another clade within Spilomelinae in the BI analysis.

The phylogenetic trees of 40 pyraloid mitogenomes were fully resolved with identical topology, except for the ‘non-PS clade’ (Regier et al. [8]) that includes Odontiinae based on PCG123, PCG12 and AA for both BI and ML analyses (Figure 5, Figure 6 and Figure 7; Tables S4 and S5). In all of the phylogenetic trees obtained, we think that the topology of the AA tree is optimal, which is congruent with the previous results of nuclear genes. In all trees based on three datasets, the monophyly of the two families, Pyralidae and Crambidae, was well supported, as has been indicated with the morphology-based and the molecular-based results [1,4,8,68].

Within the Pyralidae, all analyses consistently supported its monophyly of three of the subfamilies, i.e., Pyralinae, Galleriinae and Phycitinae, and the inclusion of the subfamilies, i.e., Pyralinae, Galleriinae, Epipaschiinae and Phycitinae, with the exception of the Chrysauginae, that was not included for a lack of online mitogenomic data. The relationship of the four subfamilies was recovered as Galleriinae + (Phycitinae + (Pyralinae + Epipaschiinae)), which is compatible with previous studies based on mitogenomic data or multiple gene markers [4,8,20]. The subfamily Galleriinae was placed as the sister group to the other three subfamilies with very strong support (PP = 1, BS = 100) (Figure 5, Figure 6 and Figure 7).

As for the Crambidae, our results show a basal split into two sister lineages, one consisting of Pyraustinae and Spilomelinae, and another comprising the remaining six subfamilies of Crambidae included in this study (Acentropinae, Crambinae, Glaphyriinae, Odontiinae, Schoenobiinae and Scopariinae). The two lineages generally correspond to the ‘PS clade’ and ‘non-PS clade’ as defined by Regier et al. [8] based on nuclear genes. Within the ‘PS clade’, our analyses confirm the sister relationship of Pyraustinae and Spilomelinae with very strong support (PP = 1, BS = 100), which is compatible with previous studies [4,20,59,69].

In this study, the ‘non-PS clade’ included six subfamilies, and a subset of the currently recognized subfamilies in this taxon: Acentropinae, Crambinae, Glaphyriinae, Odontiinae, Schoenobiinae, Scopariinae [4,8]. In our study, the ‘non-PS clade’ was clustered into two branches, which corresponds to the ‘OG clade’ and ‘CAMMSS clade’ according to Regier et al. [8], respectively. In the ‘OG clade’, all the results based on PCG123, PCG12 and AA for BI and ML analyses supported the monophyly of the Odontiinae, however, the Glaphyriinae was recovered as paraphyletic based on PCG12 and PCG123, and as monophyly with moderate (PP = 0.5) or high support values (BS = 87) based on AA, which was consistent with the previous studies [4,8].

Within the ‘non-PS clade’, our results based on three datasets exhibited different topologies, and the inconsistence mainly focused on the ‘CAMMSS clade’. The four subfamilies in this study were recovered as Acentropinae + (Scopariinae + (Crambinae + Schoenobiinae)) based on the PCG123 in both BI and ML analyses (Figure 5). This confirmed the mitogenome-based results of Zhu et al. [18], which were recovered as Acentropinae + (Crambinae + Schoenobiinae) based on the PCG123 and PCG12 in BI and ML analyses, despite the fact that Scopariinae was not involved. Moreover, in our result, the relationships Scopariinae + (Schoenobiinae + (Acentropinae + Crambinae)) and Schoenobiinae + (Scopariinae + (Acentropinae + Crambinae)) were recovered in BI and ML analyses based on PCG12 with low-to-moderate support, respectively (Figure 6).

As Cameron [10] mentioned, the inclusion of third codon positions may result in serious artifacts due to the faster rate of evolution, and it seems that the results of the inconsistent relationships within the ‘CAMMSS clade’ based on PCG123 and PCG12 are an example of this phenomenon. Moreover, the long branch of Schoenobiinae was very distinctive in all topologies, and it might be another reason for the discrepant topologies in different analyses. As only one species of this subfamily was sequenced, its position within the ‘CAMMSS clade’ needs extra samplings to confirm. In addition, in the ML analysis of AA (Figure 7), the relationship (Scopariinae + Crambinae) + (Acentropinae + Schoenobiinae) was recovered. It was identical to the findings of Regier et al. [8] and Léger et al. [4], which was based on several nuclear genes combined with a single mitochondrial gene COI. The BI analysis of AA (Figure 7) showed an identical topology for the ‘CAMMSS clade’ with the ML analysis, but the ‘OG clade’ was formed as polyphyletic.

On the basis of the above analyses, a close relationship between Scopariinae and Crambinae and between Schoenobiinae and Acentropinae for the ‘CAMMSS clade’ and the monophyly of Odontiinae and Glaphyriinae might be more realistic. Perhaps, more mitogenomic data of pyraloid taxa, a combination of mitogenomic and nuclear genes or even the genome-scaled analysis would help to confirm or understand the phylogenetic relationships within Pyraloidea.

## 4. Conclusions

In this study, we reported the mitogenome sequences of *Dausara latiterminalis*, *Heortia vitessoides*, and *Pseudonoorda nigropunctalis*, which are the first complete mitogenomes in the subfamily Odontiinae. Compared to other previously reported mitogenomes of Pyraloidea, the newly sequenced odontiine complete mitogenomes are conserved in gene organization, base composition, codon usage of PCGs and secondary structures of tRNAs. The phylogenetic analyses inferred from mitogenomes (PCG123, PCG12 and AA) produced a well-resolved framework for the relationships of Pyraloidea, and the monophyly and position of Odontiinae were also confirmed. Our results were largely congruent with the previous results based on nuclear and mitogenomic genes, except for the relationship within the ‘CAMMSS clade’. As for the ‘non-PS clade’, the ML analysis of the AA dataset was inconsistent with the previous studies based on mitogenomic genes but was identical with the former results based on multiple nuclear genes. The results of this study provided a new or alternative insight to improve the current phylogeny of the Pyraloidea. Moreover, it suggested that mitogenome data were useful for resolving the phylogenetic issues of Pyraloidea and the topologies generated from amino acids might be more realistic and reliable. In addition, extensive samples of multiple taxa and larger scale analyses with more genes, or even a whole genome may be helpful to reconstruct a comprehensive framework of the pyraloid phylogeny.

## Figures and Tables

**Table 1:** Mitogenomes of Pyraloidea and outgroups used in this study.

Subfamily	Taxa	GenBank Accession No.	References
Pyralidae			
Pyralinae	*Hypsopygia regina*	KP327714	Unpublished
	*Endotricha consocia*	MF568544	[18]
Phycitinae	*Meroptera pravella*	MF073207	[28]
	*Ephestia kuehniella*	KF305832	[29]
	*Amyelois transitella*	KT692987	[30]
	*Ephestia kuehniella*	KU877167	Unpublished
	*Plodia interpunctella*	KP729178	[31]
Galleriinae	*Galleria mellonella*	KT750964	[32]
	*Corcyra cephalonica*	HQ897685	[33]
Epipaschiinae	*Lista haraldusalis*	KF709449	[34]
Crambidae			
Odontiinae	*Dausara latiterminalis*	NW732137	This study
	*Heortia vitessoides*	NW732138	This study
	*Pseudonoorda nigropunctalis*	NW732139	This study
Crambinae	*Chilo suppressalis*	HQ860290	[35]
	*Chilo sacchariphagus*	KU188518	Unpublished
	*Diatraea saccharalis*	FJ240227	[36]
Pyraustinae	*Loxostege sticticalis*	KR080490	Unpublished
	*Ostrinia furnacalis*	AF467260	[37]
	*Ostrinia nubilalis*	AF442957	[37]
Schoenobiinae	*Scirpophaga incertulas*	KC493629	[38]
	*Scirpophaga incertulas*	KF751706	Unpublished
Acentropinae	*Elophila interruptalis*	KC894961	[39]
	*Parapoynx crisonalis*	KT443883	Unpublished
	*Paracymoriza prodigalis*	JX144892	[40]
	*Paracymoriza distinctalis*	KF859965	[41]
Spilomelinae	*Glyphodes quadrimaculalis*	KF234079	[42]
	*Pycnarmon lactiferalis*	KX426346	[43]
	*Tyspanodes hypsalis*	KM453724	[44]
	*Glyphodes pyloalis*	KM576860	Unpublished
	*Tyspanodes striata*	KP347977	Unpublished
	*Cnaphalocrocis medinalis*	JN246082	[45]
	*Cnaphalocrocis medinalis*	JQ647917	[46]
	*Dichocrocis punctiferalis*	JX448619	[47]
	*Haritalodes derogata*	KR233479	[48]
	*Maruca vitrata*	KP327715	Unpublished
	*Nomophila noctuella*	KM244688	[49]
	*Spoladea recurvalis*	KJ739310	[50]
	*Tyspanodes striata*	KP347977	Unpublished
Glaphyriinae	*Evergestis junctalis*	KP347976	Unpublished
Scopariinae	*Eudonia angustea*	KJ508052	[14]
Alucitidae	*Alucita montana*	KJ508059	[14]
Pterophoridae	*Emmelina monodactyla*	KJ508063	[14]
Thyrididae	*Pyrinioides aurea*	KT337662	[25]
Zygaenidae	*Phauda flammans*	NC_047243	[51]
Zygaenidae	*Rhodopsona rubiginosa*	NC_025761	[49]

**Table 2:** Organization of the mitogenomes of *D. latiterminalis*, *H. vitessoides* and *P. nigropunctalis*.

Feature	Strand	Position(from)	Position(to)	Length	Intergenic Nucleotides	Anticodon	Initial Codon	Stop Codon
trnM	J	1/1/1	69/68/66	69/68/66	0/0/0	CAT		
trnI	J	70/69/67	133/132/130	64/64/64	−3/−3/−3	GAT		
trnQ	N	131/130/128	199/198/196	69/69/69	0/0/0	TTG		
ND2	J	200/199/197	1213/1212/1210	1014/1014/1014	0/0/4		ATT/ATC/ATT	TAA/TAA/TAA
trnW	J	1214/1213/1215	1280/1279/1281	67/67/67	−8/−8/−8	TCA		
trnC	N	1273/1272/1274	1339/1338/1337	67/67/64	0/0/0	GCA		
trnY	N	1340/1339/1338	1406/1405/1402	67/67/65	−8/8/0	GTA		
COX1	J	1399/1414/1403	2949/2952/2941	1551/1539/1539	−5/−5/−5		ATT/TTG/TTG	TAA/TAA/TAA
trnL	J	2945/2948/2937	3011/3014/3003	67/67/67	0/0/0	TAA		
COX2	J	3012/3015/3004	3693/3696/3720	682/682/717	0/0/−35		ATG/ATG/ATG	T/T/TAA
trnK	J	3694/3697/3686	3763/3767/3755	70/71/70	0/1/0	CTT		
trnD	J	3764/3769/3756	3829/3838/3821	66/70/66	0/0/0	GTC		
ATP8	J	3830/3839/3822	3991/4000/3983	162/162/162	−7/−7/−7		ATT/ATT/ATT	TAA/TAA/TAA
ATP6	J	3985/3994/3977	4665/4674/4657	681/681/681	−1/−1/−1		ATG/ATG/ATG	TAA/TAA/TAA
COX3	J	4665/4674/4657	5453/5462/5445	789/789/789	2/3/2		ATG/ATG/ATG	TAA/TAA/TAA
trnG	J	5456/5466/5448	5521/5532/5514	66/67/67	0/0/0	TCC		
ND3	J	5522/5533/5515	5875/5886/5868	354/354/354	3/7/7		ATT/ATT/ATT	TAA/TAA/TAA
trnA	J	5879/5894/5876	5944/5958/5940	66/65/65	0/2/0	TGC		
trnR	J	5945/5961/5941	6007/6025/6003	63/65/63	0/4/0	TCG		
trnN	J	6008/6030/6004	6073/6096/6069	66/67/66	0/0/0	GTT		
trnS	J	6074/6097/6070	6139/6162/6135	66/66/66	1/0/1	GCT		
trnE	J	6141/6163/6137	6206/6228/6201	66/66/65	−2/12/4	TTC		
trnF	N	6205/6241/6206	6270/6308/6270	66/68/65	4/−1/−17	GAA		
ND5	N	6275/6308/6254	8008/8043/8005	1734/1736/1752	0/0/0		ATT/ATT/ATT	TAA/TTA/TAA
trnH	N	8009/8044/8006	8074/8109/8072	66/66/67	−1/0/9	GTG		
ND4	N	8074/8110/8082	9415/9453/9422	1342/1344/1341	17/2/3		ATG/ATG/ATG	TAA/TAA/TAA
ND4L	N	9433/9456/9426	9723/9749/9716	291/294/291	2/2/5		ATG/ATG/ATA	TAA/TAA/TAA
trnT	J	9726/9752/9722	9790/9818/9786	65/67/65	0/0/0	TGT		
trnP	N	9791/9819/9787	9856/9883/9852	66/65/66	0/0/0	TGG		
ND6	J	9857/9884/9853	10,390/10,465/10,386	534/582/534	4/0/−1		ATA/ATC/ATA	TAA/TAA/TAA
CYTB	J	10,395/10,466/10,386	11,543/11,620/11,531	1149/1155/1146	−2/3/4		ATG/ATG/ATG	TAA/TAA/TAA
trnS	J	11,542/11,624/11,536	11,609/11,688/11,600	68/65/65	16/16/3	TGA		
ND1	N	11,626/11,705/11,604	12,564/12,643/12,554	939/939/951	1/1/0		ATG/ATG/ATG	TAA/TAA/TAG
trnL	N	12,566/12,645/12,555	12,633/12,712/12,621	68/68/67	−25/−19/−23	TAG		
l-rRNA	N	12,609/12,694/12,599	13,977/14,057/13,967	1369/1364/1369	−12/−12/−12			
trnV	N	13,966/14,046/13,956	14,030/14,111/14,021	65/66/66	−1/−1/−1	TAC		
s-rRNA	N	14,030/14,111/14,021	14,812/14,890/14,804	783/780/784	0/0/0			
A + T-rich region		14,901/15,057/14,891	15,235/15,404/15,170	334/348/280				

## Data Availability

All sequences were deposited in the GenBank under accession numbers of NW732137–NW732139.

## Supplementary Materials

The following are available online at https://www.mdpi.com/article/10.3390/insects12060486/s1. Table S1. Collecting information of specimens in present study. Table S2. The best fit DNA model of each locus in datasets PCG123 and PCG12 selected by jModeltest 2.1.7. Table S3. Nucleotide composition of mitochondrial genomes of three odontiine species. Table S4. Phylogenetic relationships among pyraloid species by BI analysis based on the PCG12 dataset. Table S5. Phylogenetic relationships among pyraloid species by BI analysis based on the AA dataset.

## References

[1] Munroe E., Solis M.A., Kristensen N. (1999). Pyraloidea. Lepidoptera, Moths and Butterflies, Volume 1: Evolution, Systematics, and Biogeography. Handbook of Zoology.

[2] Solis M.A. (2007). Phylogenetic studies and modern classification of the Pyraloidea (Lepidoptera). Rev. Colomb. Entomol..

[3] Hayden J.E. (2011). Revision of *Cliniodes* Guenée (Lepidoptera: Crambidae: Odontiinae). Ann. Carnegie Mus..

[4] Léger T., Mally R., Neinhuis C., Nuss M. (2021). Refining the phylogeny of Crambidae with complete sampling of subfamilies (Lepidoptera, Pyraloidea). Zool. Scr..

[5] Nuss M., Landry B., Mally R., Vegliante F., Tränkner A., Bauer F., Hayden J., Segerer A., Schouten R., Li H. Global Information System on Pyraloidea. http://www.pyraloidea.org.

[6] Chen Z., Li D., Wang L., Li Y., Huang X., Qin C. (2011). Studies on biological characteristics of *Heortia vitessoides* Moore on *Aquilaris sinensis*. China Plant Protect..

[7] Zhang J., Zhang R., Zeng H., Zhang Z., Wu Q. (2009). Study on biological characteristics and occurrence regularity of *Pseudonoorda minor*. J. Southwest For. Univ..

[8] Regier J., Mitter C., Solis M., Hayden J., Landry B., Nuss M., Simonsen T., Yen S.-H., Zwick A., Cummings M. (2012). A molecular phylogeny for the pyraloid moths (Lepidoptera: Pyraloidea) and its implications for higher-level classification. Syst. Entomol..

[9] Boore J.L. (1999). Animal mitochondrial genomes. Nucleic Acids Res..

[10] Cameron S.L. (2014). Insect mitochondrial genomics: Implications for evolution and phylogeny. Annu. Rev. Entomol..

[11] Curole J.P., Kocher T.D. (1999). Mitogenomics: Digging deeper with complete mitochondrial genomes. Trends Ecol. Evol..

[12] Simon C., Buckley T.R., Frati F., Stewart J.B., Beckenbach A.T. (2006). Incorporating molecular evolution into phylogenetic analysis, and a new compilation of conserved polymerase chain reaction primers for animal mitochondrial DNA. Annu. Rev. Ecol. Evol. Syst..

[13] Wang I.J. (2010). Recognizing the temporal distinctions between landscape genetics and phylogeography. Mol. Ecol..

[14] Timmermans M.J.T.N., Lees D.C., Simonsen T.J. (2014). Towards a mitogenomic phylogeny of Lepidoptera. Mol. Phylogenet. Evol..

[15] Li H., Leavengood J.M., Chapman E.G., Burkhardt D., Song F., Jiang P., Liu J., Zhou X., Cai W. (2017). Mitochondrial phylogenomics of Hemiptera reveals adaptive innovations driving the diversification of true bugs. Proc. R. Soc. B.

[16] Chen S., Li F., Lan X., You P. (2017). Complete mitochondrial genomes of three Spilomelinae species and a preliminary phylogenetic analysis of the Pyraloidea (Insecta: Lepidoptera). Chin. J. Appl. Entomol..

[17] Chen S., Zhang H., Wu S., You P. (2018). Sequencing and analysis of the complete mitochondrial genome of *Diaphania perspectalis* and *Pleuroptya inferior* (Insecta: Lepidoptera). J. Shaanxi Norm. Univ..

[18] Zhu W., Yan J., Song J., You P. (2018). The first mitochondrial genomes for Pyralinae (Pyralidae) and Glaphyriinae (Crambidae), with phylogenetic implications of Pyraloidea. PLoS ONE.

[19] Liu Q., Jiang X., Hou X., Yang H., Chen W. (2018). The mitochondrial genome of *Ephestia elutella* (Insecta: Lepidoptera: Pyralidae). Mitochondrial DNA Part B.

[20] Yang M., Song L., Mao J., Shi Y., Wu C., Zhang Y., Huang L., Peng W., Liu X. (2018). Complete mitochondrial genome of the soybean leaffolder, *Omiodes indicata* (Lepidoptera: Pyraloidea: Crambidae), and phylogenetic analysis for Pyraloidea. Int. J. Biol. Macromol..

[21] Yang L., Dai J., Gao Q., Yuan G., Liu J., Sun Y., Wang L., Qian C., Zhu B., Liu C. (2020). Characterization of the complete mitochondrial genome of *Orthaga olivacea* Warre (Lepidoptera Pyralidae) and comparison with other Lepidopteran insects. PLoS ONE.

[22] Meng G., Li Y., Yang C., Liu S. (2019). MitoZ: A toolkit for animal mitochondrial genome assembly, annotation and visualization. Nucleic Acids Res..

[23] Zhang X., Tang L., Chen J., You P. (2020). The complete mitochondrial genome of *Amesia sanguiflua* (Lepidoptera, Zygaenidae). Mitochondrial DNA Part B.

[24] Qin J., Li J., Gao Q., Wilson J.J., Zhang A. (2019). Mitochondrial phylogeny and comparative mitogenomics of closely related pine moth pests (Lepidoptera: Dendrolimus). PeerJ.

[25] Zhu W., You P. (2015). Complete mitochondrial genome of *Camptochilus aurea* (Lepidoptera: Thyrididae). Mitochondrial DNA.

[26] Tamura K., Peterson D., Peterson N., Stecher G., Nei M., Kumar S. (2011). MEGA5: Molecular evolutionary genetics analysis using maximum likelihood, evolutionary distance, and maximum parsimony methods. Mol. Biol. Evol..

[27] Perna N.T., Kocher T.D. (1995). Patterns of nucleotide composition at fourfold degenerate sites of animal mitochondrial genomes. J. Mol. Evol..

[28] Ali M., Almaden J., Balchan N., Bennici C.E., Bhasin J., Brown C., Carlson H., Chavda A., Deckert J., Eastman T. (2017). The complete mitochondrial genome of the lesser aspen webworm moth *Meroptera pravella* (Insecta: Lepidoptera: Pyralidae). Mitochondrial DNA Part B.

[29] Traut W., Vogel H., Glöckner G., Hartmann E., Heckel D.G. (2013). High-throughput sequencing of a single chromosome: A moth W chromosome. Chromosome Res..

[30] Chang Z., Shen Q. (2016). The complete mitochondrial genome of the navel orangeworm *Amyelois transitella* (Insecta: Lepidoptera: Pyralidae). Mitochondrial DNA Part A.

[31] Liu Q., Chai X., Bian D., Zhou C., Tang B. (2015). The complete mitochondrial genome of *Plodia interpunctella* (Lepidoptera: Pyralidae) and comparison with other Pyraloidea insects. Genome.

[32] Park Y., Park C., Hong S., Jung B., Ibal J.C., Park G., Shin J. (2017). The complete mitochondrial genome sequence of the greater wax moth *Galleria mellonella* (Insecta, Lepidoptera, Pyralidae): Sequence and phylogenetic analysis comparison based on whole mitogenome. Mitochondrial DNA Part B.

[33] Wu Y., Li J., Zhao J., Su T., Luo A., Fan R., Chen M., Wu C., Zhu C. (2012). The complete mitochondrial genome of the rice moth, *Corcyra cephalonica*. J. Insect Sci..

[34] Ye F., Yu H., Li P., You P. (2015). The complete mitochondrial genome of *Lista haraldusalis* (Lepidoptera: Pyralidae). Mitochondrial DNA.

[35] Yin J., Wang A., Hong G., Cao Y., Wei Z. (2011). Complete mitochondrial genome of *Chilo suppressalis* (Walker) (Lepidoptera: Crambidae). Mitochondrial DNA.

[36] Yin Y., Qu F., Yang Z., Zhang X., Yue B. (2014). Structural characteristics and phylogenetic analysis of the mitochondrial genome of the rice leafroller, *Cnaphalocrocis medinalis* (Lepidoptera: Crambidae). Mol. Biol. Rep..

[37] Coates B.S., Sumerford D.V., Hellmich R.L., Lewis L.C. (2005). Partial mitochondrial genome sequences of *Ostrinia nubilalis* and *Ostrinia furnicalis*. Int. J. Biol. Sci..

[38] Cao S., Yu W., Sun M., Du Y. (2014). Characterization of the complete mitochondrial genome of *Tryporyza incertulas*, in comparison with seven other Pyraloidea moths. Gene.

[39] Park J., Kim M., Kim S., Kim I. (2014). Complete mitochondrial genome of an aquatic moth, *Elophila interruptalis* (Lepidoptera: Crambidae). Mitochondrial DNA.

[40] Ye F., Shi Y., Xing L., Yu H., You P. (2013). The complete mitochondrial genome of *Paracymoriza prodigalis* (Leech, 1889) (Lepidoptera), with a preliminary phylogenetic analysis of Pyraloidea. Aquat. Insects.

[41] Ye F., You P. (2016). The complete mitochondrial genome of *Paracymoriza distinctalis* (Lepidoptera: Crambidae). Mitochondrial DNA Part A.

[42] Park J., Kim M., Ahn S., Kim I. (2015). Complete mitochondrial genome of the grass moth *Glyphodes quadrimaculalis* (Lepidoptera: Crambidae). Mitochondrial DNA.

[43] Chen S., Li F., Lan X., You P. (2016). The complete mitochondrial genome of *Pycnarmon lactiferalis* (Lepidoptera: Crambidae). Mitochondrial DNA Part B.

[44] Wang J., Li P., You P. (2016). The complete mitochondrial genome of *Tyspanodes hypsalis* (Lepidoptera: Crambidae). Mitochondrial DNA Part A.

[45] Chai H., Du Y., Zhai B. (2012). Characterization of the complete mitochondrial genomes of *Cnaphalocrocis medinalis* and *Chilo suppressalis* (Lepidoptera: Pyralidae). Int. J. Biol. Sci..

[46] Wan X., Kim M., Kim I. (2013). Description of new mitochondrial genomes (*Spodoptera litura*, Noctuoidea and *Cnaphalocrocis medinalis*, Pyraloidea) and phylogenetic reconstruction of Lepidoptera with the comment on optimization schemes. Mol. Biol. Rep..

[47] Wu Q., Gong Y., Shi B., Gu Y., Wei S. (2013). The complete mitochondrial genome of the yellow peach moth *Dichocrocis punctiferalis* (Lepidoptera: Pyralidae). Mitochondrial DNA.

[48] Zhao J., Sun Y., Xiao L., Tan Y., Bai L. (2016). Complete mitochondrial genome of Cotton Leaf Roller *Haritalodes derogata* (Lepidoptera: Crambidae). Mitochondrial DNA Part A.

[49] Tang M., Tan M., Meng G., Yang S., Su X., Liu S., Song W., Li Y., Wu Q., Zhang A. (2014). Multiplex sequencing of pooled mitochondrial genomes—A crucial step toward biodiversity analysis using mito-metagenomics. Nucleic Acids Res..

[50] He S., Zou Y., Zhang L., Ma W., Zhang X., Yue B. (2015). The complete mitochondrial genome of the beet webworm, *Spoladea recurvalis* (Lepidoptera: Crambidae) and its phylogenetic implications. PLoS ONE.

[51] Zhang R., Li J., Geng S., Yang J., Zhang X., An Y., Li C., Cui H., Li X., Wang Y. (2020). The first mitochondrial genome for Phaudidae (Lepidoptera) with phylogenetic analyses of Zygaenoidea. Int. J. Biol. Macromol..

[52] Darriba D., Taboada G.L., Doallo R., Posada D. (2012). jModelTest 2: More models, new heuristics and parallel computing. Nat. Methods.

[53] Ronquist F., Teslenko M., van der Mark P., Ayres D.L., Darling A., Hohna S., Larget B., Liu L., Suchard M.A., Huelsenbeck J.P. (2012). MrBayes 3.2: Efficient Bayesian phylogenetic inference and model choice across a large model space. Syst. Biol..

[54] Nguyen L.-T., Schmidt H.A., von Haeseler A., Minh B.Q. (2015). IQ-TREE: A fast and effective stochastic algorithm for estimating maximum-likelihood phylogenies. Mol. Biol. Evol..

[55] Lartillot N., Philippe H. (2004). A Bayesian mixture model for across-site heterogeneities in the amino-acid replacement process. Mol. Biol. Evol..

[56] Rota-Stabelli O., Yang Z., Telford M.J. (2009). MtZoa: A general mitochondrial amino acid substitutions model for animal evolutionary studies. Mol. Phylogenet. Evol..

[57] Rambaut A., Suchard M.A., Xie D., Drummond A. Tracer v1.6. http://tree.bio.ed.ac.uk/software/tracer/.

[58] Rambaut A. Figtree 1.4.3. http://tree.bio.ed.ac.uk/software/figtree/.

[59] Ma H., Zheng X., Peng M., Bian H., Chen M., Liu Y., Jiang X., Qin L. (2016). Complete mitochondrial genome of the meadow moth, *Loxostege sticticalis* (Lepidoptera: Pyraloidea: Crambidae), compared to other Pyraloidea moths. J. Asia-Pac. Entomol..

[60] Zhou N., Dong Y., Qiao P., Yang Z. (2020). Complete mitogenomic structure and phylogenetic implications of the genus *Ostrinia* (Lepidoptera: Crambidae). Insects.

[61] Ojala D., Montoya J., Attardi G. (1981). tRNA punctuation model of RNA processing in human mitochondria. Nature.

[62] Yong H., Song S., Lim P., Chan K., Chow W., Eamsobhana P. (2015). Complete mitochondrial genome of *Bactrocera arecae* (Insecta: Tephritidae) by next-generation sequencing and molecular phylogeny of Dacini tribe. Sci. Rep..

[63] Chen P., Zheng B., Liu J., Wei S. (2016). Next-generation sequencing of two mitochondrial genomes from family pompilidae (Hymenoptera: Vespoidea) reveal novel patterns of gene arrangement. Int. J. Mol. Sci..

[64] Shi Y., Chu Q., Wei D., Qiu Y., Shang F., Dou W., Wang J. (2016). The mitochondrial genome of booklouse, *Liposcelis sculptilis* (Psocoptera: Liposcelididae) and the evolutionary timescale of Liposcelis. Sci. Rep..

[65] Zhang H., Ye F. (2017). Comparative mitogenomic analyses of praying mantises (Dictyoptera, Mantodea): Origin and evolution of unusual intergenic gaps. Int. J. Biol. Sci..

[66] Lavrov D.V., Brown W.M., Boore J.L. (2000). A novel type of RNA editing occurs in the mitochondrial tRNAs of the centipede *Lithobius forficatus*. Proc. Natl. Acad. Sci. USA.

[67] Zhang D., Szymura J., Hewitt G.M. (1995). Evolution and structural conservation of the control region of insect mitochondrial DNA. J. Mol. Evol..

[68] Minet J. (1981). Les Pyraloidea et leurs principals divisions systématiques. Bull. Soc. Entomol. Fr..

[69] Léger T., Landry B., Nuss M. (2019). Phylogeny, character evolution and tribal classification in Crambinae and Scopariinae (Lepidoptera, Crambidae). Syst. Entomol..

